# Prediction of Long-Term Tensile Properties of Glass Fiber Reinforced Composites under Acid-Base and Salt Environments

**DOI:** 10.3390/polym14153031

**Published:** 2022-07-26

**Authors:** Jihua Zhu, Yangjian Deng, Piyu Chen, Gang Wang, Hongguang Min, Wujun Fang

**Affiliations:** 1 Guangdong Province Key Laboratory of Durability for Marine Civil Engineering, College of Civil and Transportation Engineering, Shenzhen University, Shenzhen 518060, China; zhujh@szu.edu.cn (J.Z.); yuangkin@126.com (Y.D.); 2 School of Civil and Environmental Engineering, Harbin Institute of Technology, Shenzhen 518055, China; 19b954012@stu.hit.edu.cn; 3 Central Research Institute of Building and Construction (Shenzhen) Co., Ltd., MCC Group, Shenzhen 518055, China; fangwujun@cribc.com

**Keywords:** GFRP, environmental degradation, tensile properties, DIC, long-term prediction

## Abstract

This study investigates the effects of deionized water, seawater, and solutions with various concentrations (5% and 10% by mass) of HCl and NaOH on the physical and mechanical properties of glass fiber reinforced polymers (GFRPs) through aging tests at 20 °C, 50 °C, and 80 °C. The tensile properties of GFRP were assessed by tensile testing at room temperature, and the strain during the tensile process was observed using digital image correlation. Additionally, the degradation mechanism was analyzed using scanning electron microscopy, and long-term tensile properties were predicted based on the Arrhenius model. The results indicated that the tensile strength of the GFRP decreased by 22%, 71%, and 87% after 56 d of exposure to 5% NaOH solutions at 20 °C, 50 °C, and 80 °C, respectively. The alkaline solutions had a more severe effect on the GFRP than deionized water, seawater, and acidic solutions. The experimental values and Arrhenius model predictions were found to be in good agreement with each other.

## 1. Introduction

Pultruded glass fiber reinforced polymers (GFRPs), with high specific strength, non-magnetic properties, and ease of forming, have the added advantage of economy over carbon and basalt fiber-reinforced composites, and they are widely used in aerospace, automotive and marine, medical, and civil engineering fields [1,2]. However, when compared with steel, pultruded GFRPs are less ductile and the failure mode of the material is usually brittle. When the GFRP is applied to critical structures, such as offshore drilling platforms, wastewater treatment plants, power plants, storage tanks, and pipelines in aggressive environments, it is often subjected to aggressive media such as seawater, high temperatures and humidity, as well as acidic and alkaline environments [3,4,5,6]. Under long-term effects of the aggressive media, the GFRP is prone to aging that leads to the degradation of the GFRP’s structural performance and causes significant social and economic loss. To better promote the application of the GFRP, it is necessary to obtain performance degradation data for the GFRP in actual service and predict its long-term performance using accelerated life characterization methods with the aim of revealing the degradation mechanism of the GFRP and its degradation law. Studies have shown that water-based solution immersion is one of the conditions that causes the greatest degradation in GFRPs [7] because water absorption by GFRP causes hydrolysis and plasticization of the resin matrix, weakening the fiber-matrix interface [8,9].

Several studies have been conducted in recent years to investigate the durability of GFRP composites in highly corrosive environments. Shao and Kouadio [10] found that the polyester matrix GFRP did not show signs of water-absorption saturation after 260 d of immersion in water at 23 °C, despite a 17.5% decrease in tensile strength. At 70 °C, its water absorption increased and then decreased, even reaching negative values, with a 56.3% decrease in tensile strength; however, the Young’s modulus of the GFRP remained almost unchanged at the two temperatures. Grammatikos et al. [11] reported similar findings and concluded that the composite increases moisture absorption and water diffusion owing to the increase in temperature, and the entire process of water diffusion does not reach saturation. Based on the Levenberg-Marquardt algorithm, Xin and Liu [12] concluded that there are three dominant factors for the change in mass due to moisture absorption: diffusion, polymer relaxation, and composite damage.

Sousa et al. [13,14] further compared the effects of salt solution and tap water on the deterioration properties of polyurethane GFRP and epoxy resin GFRP lap joints. They found that the residual strength of the GFRP joints was higher in salt water than in tap water, which is consistent with the test results [15,16]. Furthermore, a significant post-curing phenomenon was found; the maximum reduction in the ultimate load of the GFRP was 27% during the aging period of 730 d in the 20 °C water immersion tests, but at the end of the aging period, the ultimate load reached, or even slightly exceeded, the initial ultimate load. The post-curing phenomenon has also been identified in numerous studies [11,17,18,19] and is attributed to the fact that polymer systems containing styrene polyesters and vinyl esters undergo rapid local homo-polymerization during the curing process, but the composite does not achieve complete curing in a short time, which leads to further curing during the aging period.

Feng Peng et al. [18] comparatively studied the effects of a sulfuric acid solution with pH = 5 and a mass fraction of 30% on the epoxy resin GFRP at a temperature of 60 °C and found that a high-concentration sulfuric acid solution had severe corrosion effects on the GFRP. After 90 d of immersion, the bending properties of the GFRP decreased by 34% and 73%, respectively, and the hardness decreased by 6.5% and 55.6%, respectively. Kanerva et al. [20] studied the properties of vinyl ester in a sulfuric acid solution at a temperature of 90 °C, pressure of 1.5 MPa, and concentration of 50 g/l. They found that the bending stiffness of vinyl ester decreased by 22% after one year of acid leaching, but bending strength increased by 109%. Bazli et al. [21] studied and compared the degradation of the compressive properties of vinyl ester GFRP with different cross-sectional shapes under different erosion environments and found that the O-shaped cross-section was more prone to erosion than the I- and C-shaped cross-sections. Additionally, alkali was found to cause the most serious deterioration in GFRPs. After 147 d of exposure to an alkali solution with pH = 13.6 at 20 °C, compressive strength decreased by as much as 46%. Xue et al. [22] further found that the compressive strength of the GFRP decreased faster than the tensile strength in the alkali solution. In addition, Hota et al. [19] found that the interlaminar shear stress decay of a vinyl ester GFRP plate in an alkaline environment was equivalent to that of a sample cut from the plate, indicating that there was no size effect during degradation.

To further study the deterioration law of pultruded GFRP profiles in the acid-base and salt environments, the effects of different chemical media (deionized water, artificial seawater, hydrochloric acid solution, and sodium hydroxide solution of different concentrations) at 20 °C, 50 °C, and 80 °C on the deterioration performance of the pultruded GFRP profiles were experimentally studied. Subsequently, the quality and appearance changes, tensile properties, and micro morphology of the cross section of the GFRP were analyzed. The global tensile-strain field in the fracture process was analyzed using the digital image correlation (DIC) technique. Finally, the long-term tensile properties of the pultruded GFRP profiles were predicted using the Arrhenius model.

## 2. Materials and Methods

### 2.1. Materials

**Figure 1:**
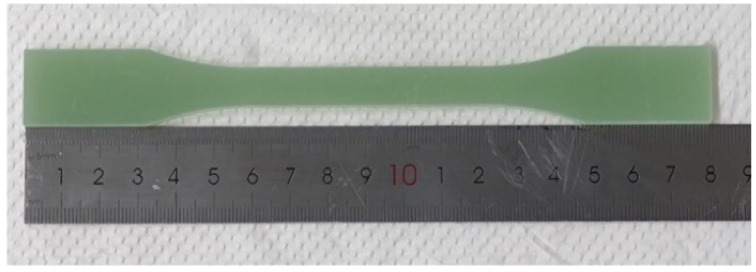
The specimen.

A GFRP plate pultruded by an epoxy resin and E-glass fiber produced by Mushi Composite Materials Technology (Suzhou) Co., Ltd. (Suzhou, China) was selected as the raw material. The glass fiber was orthogonally braided and its mass fraction was 60%. To avoid stress concentration of the specimen during the tensile test, owing to the discontinuous notch formed by manual and mechanical cutting of the GFRP plate, the GFRP specimens used in the test were cut from the GFRP plate parallel to the fiber direction using laser-cutting technology. The specimen was cut into an I-shape, referring to *GB/T 1447-2005 Fiber-reinforced plastics composites determination of tensile properties*, with a nominal thickness of approximately 4 mm and length of 180 mm, as shown in Figure 1.

### 2.2. Aging Test

**Figure 2:**
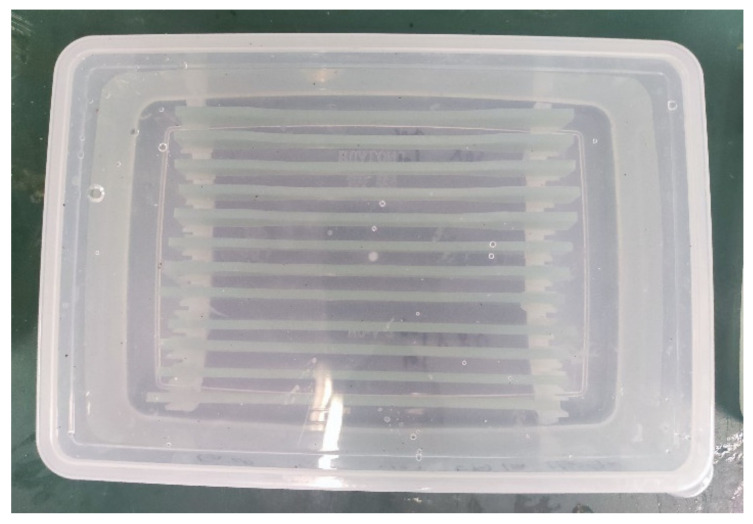
GFRP specimen immersion test.

The aging experiment explored and compared the degradation effects of four chemical media, namely deionized water (DW), artificial seawater (SW), sodium hydroxide solution (SH), and hydrochloric acid solution (HA), at different temperatures and ages on the GFRP in order to evaluate the law and mechanism of deterioration. In this test, acid and alkali solutions with mass fractions of 5% and 10% were used to further explore the degradation law of the GFRP under different concentrations of acid and alkali solutions. For the composition standard of artificial seawater, refer to *GB/T 3857-2017 Test method for chemical medium resistance of glass fiber reinforced thermosetting plastics*. The acid solution was prepared using concentrated hydrochloric acid at a concentration of 33% by mass, and the alkaline solution was prepared using analytically pure sodium hydroxide. Table 1 presents the test protocol for the deterioration tests. The GFRP specimens were soaked in six types of chemical media at three temperature gradients (20 °C, 50 °C, and 80 °C) with age settings of 28 d and 56 d. The specimens were numbered according to the following rules: the part before “-” indicates the immersion solution; 05 and 10 indicate the 5% and 10% mass percentage concentrations, respectively; the part after “-” indicates the immersion temperature. For example: 05SH-80, implies that the specimen was immersed in a 5% sodium hydroxide solution at an immersion temperature of 80 °C. The specimens were clamped and immersed in an acid-resistant, alkali-resistant, and high-temperature resistant airtight plastic box, as shown in Figure 2. Eighteen sets of test conditions were set, with 5 parallel specimens per set of conditions for a total of 180 specimens (=5 parallel specimens × 6 types of chemical media × 3 test temperatures × 2 ages). All specimens with an exposure temperature of 20 °C were placed in a cabinet at a temperature setting of 20 ± 0.5 °C, whereas the specimens with 50 °C and 80 °C conditions were placed in a water bath for heating.

### 2.3. Mass Changes

The specimens were cleaned with running water, dried before and after the deterioration test, removed, and cooled to room temperature. The mass of the specimen was then measured (the mass of the specimen before and after exposure was noted as *m*_0_ and *m_t_*, respectively, accurate to ±1 mg). The rate of mass change (*M*) was calculated using Equation (1).

(1) $$M=\frac{m_{t}-m_{0}}{m_{0}}\times 100\%$$

### 2.4. Mechanical Testing

**Figure 3:**
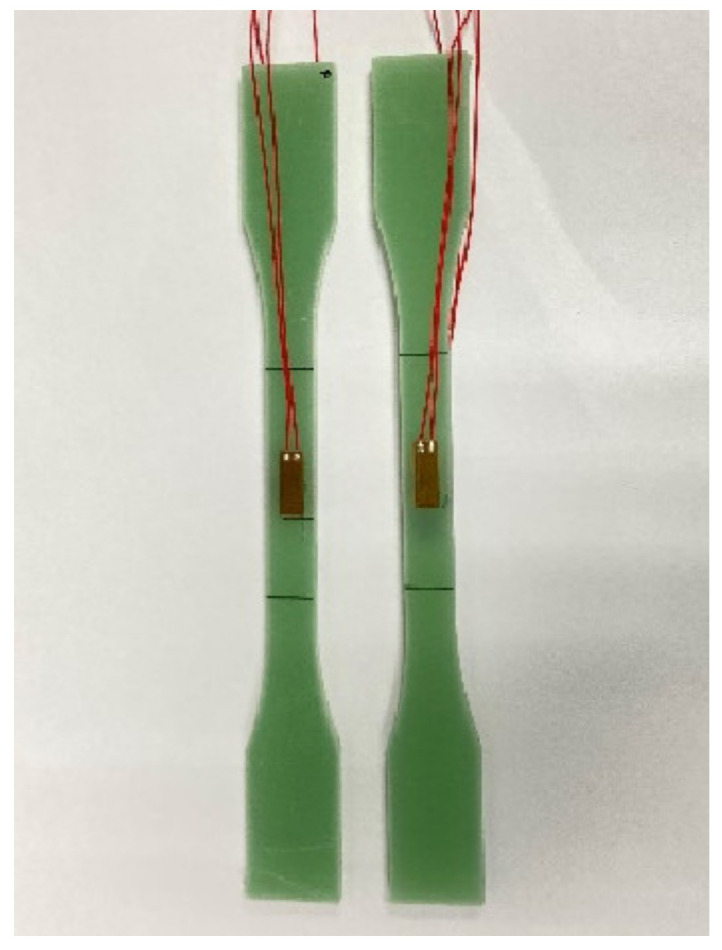
Specimens.

**Figure 4:**
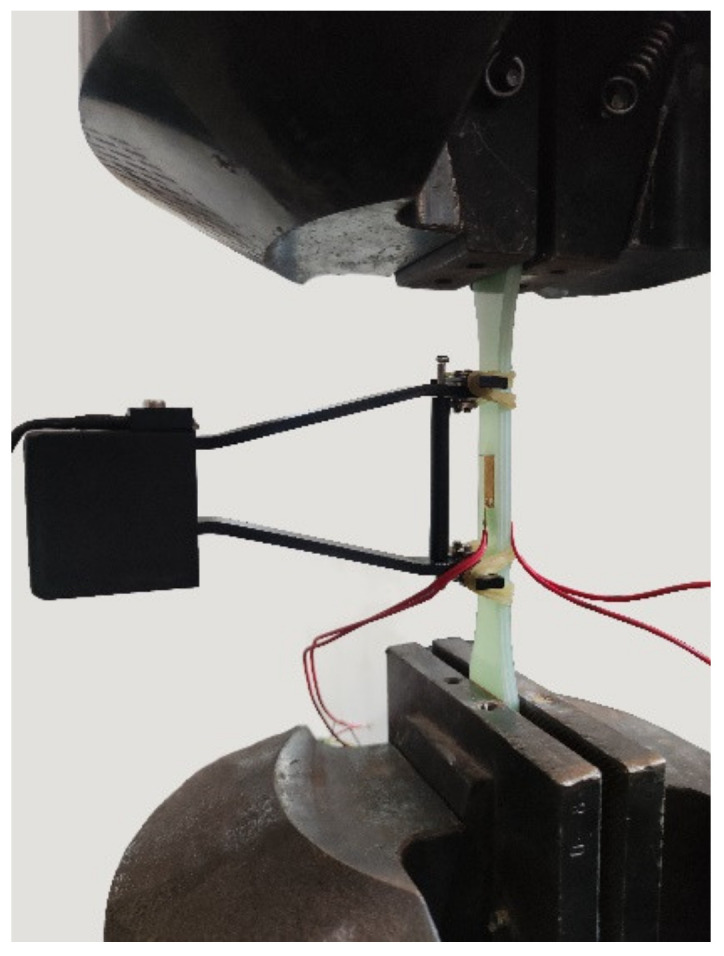
Test setup.

After drawing auxiliary lines for each tested piece, a mechanical properties test was performed. The test apparatus were selected from the PWS-50 electro-hydraulic servo dynamic tester, and the tensile test at room temperature was performed at a constant speed of 0.2 mm/min with reference to *GB/T 1447-2005 Fiber-reinforced plastics composites determination of tensile* properties. Figure 3 and Figure 4 show the test specimen and test setup with fixtures for the tensile test, respectively. Strain gauges were attached to both sides of the middle parallel section of each specimen parallel to the direction of tension and aligned centrally to obtain more accurate strain data in the initial stages of tension. An extensometer was used to indirectly measure strain over the entire scale.

### 2.5. Digital Image Correlation

DIC is an optical metrology technique, based on digital image processing and numerical computation, which provides direct full-field displacement and strain with sub-pixel accuracy by comparing digital images of the test-object surface acquired before and after deformation [23]. To obtain a more visual and comprehensive understanding of the damage process in the GFRP during tension and deformation of specimens in various regions, one of the parallel specimens in each group was subjected to tensile testing using the DIC equipment. The DIC camera used was a Pointgrey-12.3 M, with a 4096 × 3000 resolution and a lens focal length of 50 mm; the acquisition interval was set to 400 ms. Before the test was performed, the specimens were marked with a scattering of black dots on a white background to allow the computer to better distinguish the movement of the scattering and to accurately analyze the displacement of the specimen.

### 2.6. Scanning Electron Microscopy

A Phenom Pure scanning electron microscope (SEM) was used to obtain a reasonable explanation of the test phenomena from a microscopic perspective. As the subject of this test was a non-conductive material requiring a carbon or metal coating, the SEM specimen was first sprayed with a 9–12 nm platinum metal coating using sputter coating equipment and then placed in a sample cup to capture microscopic images.

## 3. Results and Discussion

### 3.1. Appearance

**Figure 5:**
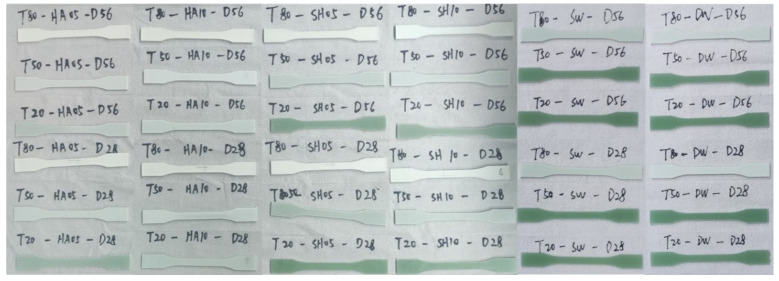
Appearance of specimens after immersion.

All specimens were bright green before the test; however, after the test, the appearance of the specimens changed, as shown in Figure 5. For the specimens tested at 20 °C, the appearance remained largely unchanged; for the specimens tested at 50 °C, the color faded from bright to light green; and for the specimens tested at 80 °C, the color degradation was more significant, with the surface resin matrix dissolving and essentially turning white. Additionally, the appearance of the specimens at different ages under the same conditions was slightly different, with the specimens showing a more pronounced color change at 56 d than at 28 d; the appearance of the specimens immersed in different chemical media under the same test conditions was also slightly different, with the specimens immersed in the sodium hydroxide solution showing the most pronounced change in appearance. The microstructural properties and chemical reactions of the samples are discussed further in this paper.

### 3.2. Mass Changes

**Figure 6:**
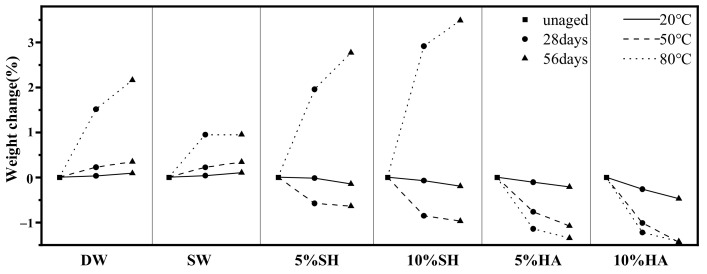
Change in specimen mass under various conditions.

Figure 6 shows the variation in the mass of the GFRP specimens over time for different hygrothermal environments. In general, mass absorption or loss under different degradation conditions increases with time; however, the rate of change can decrease or even tend to saturate. The change in mass is determined by a combination of the following: (i) water uptake, (ii) leaching of small molecular weight polymers, and (iii) leaching of dissolved hydrolysis products [4,5]. For specimens in the same chemical environment and exhibiting a consistent temperature effect, the degree of mass change increased with increasing immersion temperature. The masses increased by 0.09%, 0.34%, and 2.16% after 56 d of immersion in deionized water at 20 °C, 50 °C, and 80 °C, respectively. This was possibly because of the accelerated chemical reaction rate, owing to the increase in temperature, and a higher void pressure, resulting from the increased gas volume within the GFRP. This increase in pressure favors the extension of microcracks, thus increasing the free volume, within the specimen, that can be filled by the surrounding solution, such that the dendritic matrix absorbs more moisture [24].

In addition, it can be observed that the difference in mass change rates in the specimens, between the cases of deionized water and artificial seawater environments at 20 °C and 50 °C, is not significant; however, at 80 °C, the deionized water specimens absorb much more moisture than the artificial seawater environment specimens (the mass changes for DW-80-56 and SW-80-56 were 2.16 and 0.95%, respectively). This effect has been reported in earlier studies [13,25] and can be attributed to the cross-linking behavior of such polymers, which act as semi-permeable membranes. In the case of corrosive environments, such as acid and alkaline solutions, the specimens mainly showed mass loss (except for 05SH-80 and 10SH-80), contrary to the results [18,21]. This is because the dominant effect of mass change in the above environments is the decomposition of part of the polymer in the dendrimer matrix, its leaching into the solution [26], and dissolution of the resin on the surface of the specimen [6]. In contrast, for specimens immersed in an alkaline environment at 80 °C, an increase in mass occurred (2.77% and 3.49% after 56 d in 05SH-80 and 10SH-80 solutions, respectively), probably owing to the increase in cavities within the laminate at higher temperatures resulting in increased water uptake [27]. The overall mass increase was observed when the weight-gain due to water absorption exceeded the mass loss due to leaching of the small molecular weight segment polymer, with the surface coating of the specimen being dissolved.

### 3.3. Tensile Properties

#### 3.3.1. Stress-Strain Curves and Failure Modes

**Figure 7:**
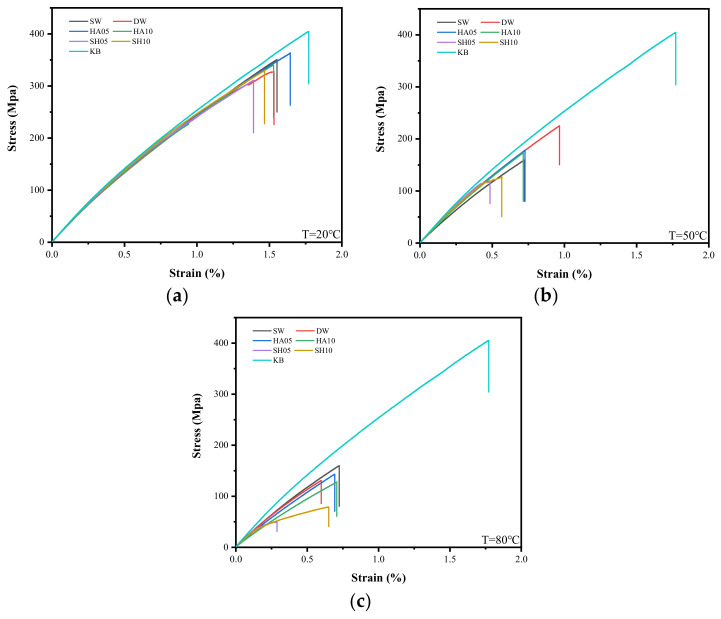
Stress-strain curves of specimens at (**a**) 20 °C, (**b**) 50 °C, and (**c**) 80 °C.

**Figure 8:**
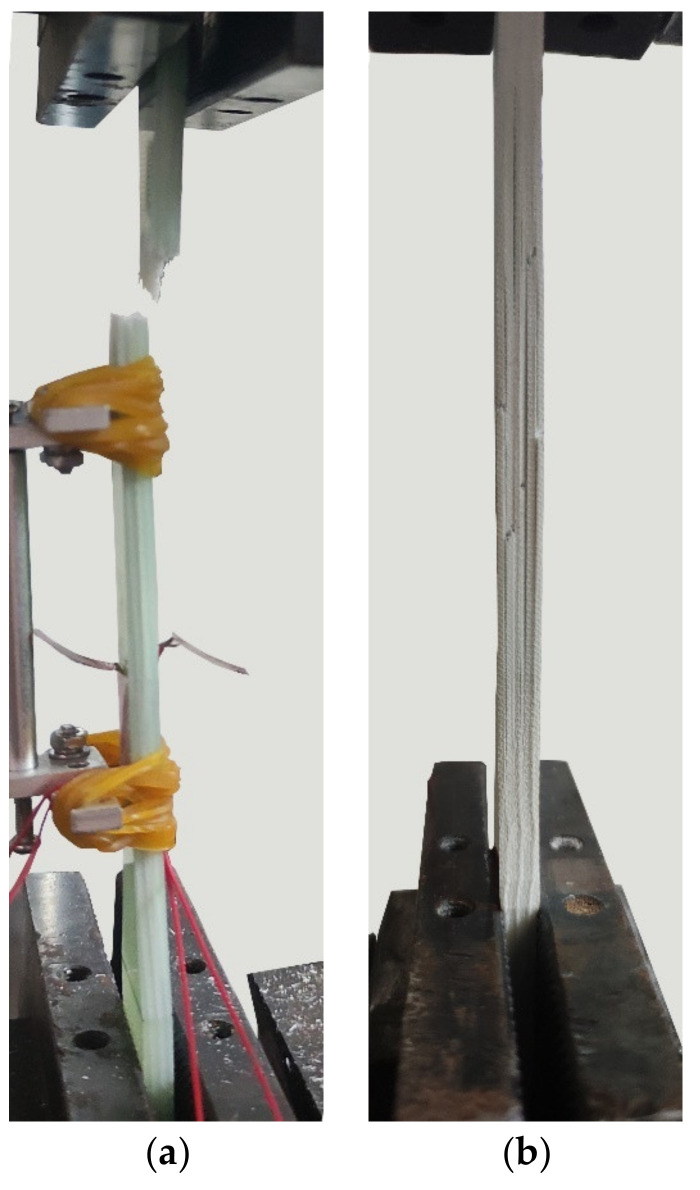
Tensile failure modes: (**a**) fiber fracture, and (**b**) delamination.

Regardless of the exposure environment, all tested specimens showed greater degradation with increases in test temperature. As shown in Figure 7, the stress-strain relationship of various specimens exposed to 20 °C was similar, indicating that the elastic modulus of the tested GFRP specimen hardly decayed at 20 °C. With the increase in immersion temperature, the stress-strain curves of the tested specimens deviate from the control specimen and the maximum stress and strain decrease, indicating that the deterioration of the specimens is aggravated by the increase in temperature, especially for the specimen immersed in the 80 °C alkaline environment. Almost all the tested specimens exhibited failure near the clamping end (as shown in Figure 8a), which was mainly due to the stress concentration caused by the variable section near the clamping end of the specimen. However, the specimens immersed in acid at 50 °C and 80 °C exhibited delamination failure (Figure 8b). This is mainly because the binder resin is much worse than the fiber under high-temperature acid-leaching conditions, thus interlayer performance is weakened. Moreover, splitting occurs when the load reaches the limit [28].

#### 3.3.2. Tensile Strength and Modulus

**Figure 9:**
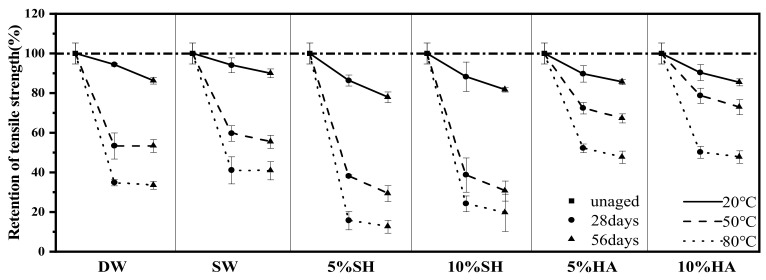
Variation of residual tensile strength as a function of immersion time and temperatures in various conditions.

The test data under all conditions are shown in Table 2. Figure 9 shows the average strength retention and error bars of the samples after 28 d and 56 d under different exposure conditions. The three exposure temperatures of 20 °C, 50 °C, and 80 °C are indicated by solid, scribed, and dotted lines, respectively, and different symbols are used for different exposure periods. Error bars indicate the magnitude of the difference between parallel samples when calculating the average strength.

As implied in Figure 9, regardless of the environment, temperature has a crucial impact on the deterioration of the specimens. Taking the sample immersed in artificial seawater as an example, after 56 d of exposure to 20 °C, the strength decreased slightly and the residual strength was 90.0%; however, when the temperature was raised to 50 °C, the deterioration was aggravated and the residual strength was 59.6%; at 80 °C, the degradation was further aggravated and the residual strength was only 40.8%. A hydrolysis reaction occurs when water comes in contact with the resin matrix. This reduces the compactness of the matrix, increases the number of pores, and reduces adhesion with the fibers, which may be the main reason for the worsening of the tensile properties of the GFRP [29]. The hydrolysis reaction between the resin matrix and water is shown in Equation (2). Hydrolysis of the resin polymer opens the ester bond and generates the corresponding carboxylic acid and free hydroxyl ions. An increase in temperature can accelerate the hydrolysis reaction, generate a higher void pressure conducive to microcrack propagation in the composite, and promote the entry of more water. However, it also affects the microstructure of the matrix, causing plasticization of the GFRP matrix [24,30] and weakening of the tensile properties of the GFRP. In addition, tensile strength does not decrease linearly with time, though most of the strength loss is generally observed after 28 d of exposure (except for DW-20), and the higher the exposure temperature, the more evident this phenomenon becomes. Taking 10SH-80 as an example, the residual tensile strength after 28 d immersion was only 24.1%, whereas the residual tensile strength after 56 d immersion was still 19.6%. It may be owing to the fact that the hydrolysis reaction of the composite is rapid, thus the rate of tensile strength loss is initially high before reducing due to the decrease in degradable reactants with the increase in degradation time. Consistent with the conclusions of many studies [7], the alkaline environment caused the most serious erosion of the GFRP. Specimens in 05SH-80-56 and 10SH-80-56 lost nearly all of their strengths, and the strength retention rates were only 12.6% and 19.6%, respectively. The tensile strength of the GFRP in deionized water and artificial seawater was lower than that of GFRP in acid. This may be because there are fewer free hydroxyl ions in the acidic environment, which inhibits the reaction between the glass fiber and hydroxyl ions to a certain extent, as shown in Equation (3). Sindhu et al. [30] suggested that fiber-matrix interface performance would be improved by immersion in an acid solution, thus improving tensile strength.

R-COO-R′ + H-OH → R-COOH + R′ + OH^−^ (2)

-Si-O-Si- + OH^−^ → Si-OH + Si-O- (3)

After 56 d of immersion in a deionized water environment at 20 °C, 50 °C, and 80 °C, the residual strengths were 86.2%, 53.3%, and 33.4%, respectively, compared to 90.0%, 55.4%, and 40.8%, respectively, for the seawater environment. Deionized water was found to reduce the tensile strength of the GFRP slightly more than artificial seawater. Compared to deionized water, the surface of the GFRP specimen is covered with a thin layer of salt in artificial seawater, which affects the exchange of matter between the interior and exterior of the specimen and, consequently, has less effect on the degradation of its tensile properties [13]. The effect of different concentrations of the sodium hydroxide solution and hydrochloric acid on the deterioration of the GFRP was also investigated. Unlike [31] and [32], it was found that a 5% sodium hydroxide solution deteriorated the GFRP more severely than the 10% solution, with the former having GFRP residual strengths of 77.9%, 29.3%, and 12.6% and the latter having the corresponding strengths of 81.7%, 30.6%, and 19.6% after 56 d of immersion at 20 °C, 50 °C, and 80 °C, respectively. One possible explanation is that, in the alkaline solution with higher concentrations of hydroxide, more hydrolysis products of the matrix and glass fibers were hoarded on the surface of the specimen at the beginning of the exposure, blocking the channels of the solution and inhibiting the matrix and glass fibers from undergoing hydrolysis reactions. In addition, it was observed that the strength degradation of the specimens immersed in 5% hydrochloric acid was slightly higher than the strength degradation of those immersed in 10% hydrochloric acid, apparently because fewer hydroxide ions inhibited the degradation of the fibers, as shown in Equation (2).

**Figure 10:**
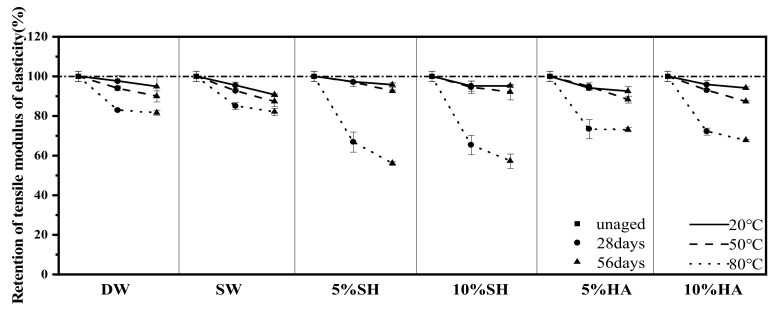
Variation of the residual tensile elastic modulus as a function of immersion time and temperatures in various conditions.

Figure 10 shows the mean modulus of elasticity retention and error bars for the specimens after 28 d and 56 d under different conditions. The pattern of stiffness change was generally consistent with that of strength change. The 20 °C and 50 °C environments had less of an effect on the stiffness of the GFRP, with the remaining stiffnesses after 56 d of exposure for DW-50, SW-50, 05SH-50, 10SH-50, 05HA-50, and 10HA-50 being 89.9%, 87.2%, 92.5%, 92.0%, 88.3%, and 87.2%, respectively. However, when the exposure temperature was increased to 80 °C, the GFRP had severe deterioration, and the stiffness residuals after 56 d of immersion in the above environments were 81.5%, 82.0%, 56.0%, 57.2%, 73.1%, and 67.6%, respectively, which is very different from GFRP grids [33]. The Young’s modulus of FRP composites is mainly determined by the modulus of elasticity of the fibers and resin matrix as well as the corresponding volume fraction [32], and it can be deduced that the glass fibers had significant deterioration at 80 °C. Overall, when comparing various exposure environments, alkali had the most significant effect on the stiffness of the GFRP, followed by acid, deionized water, and seawater.

### 3.4. DIC Analysis

**Figure 11:**
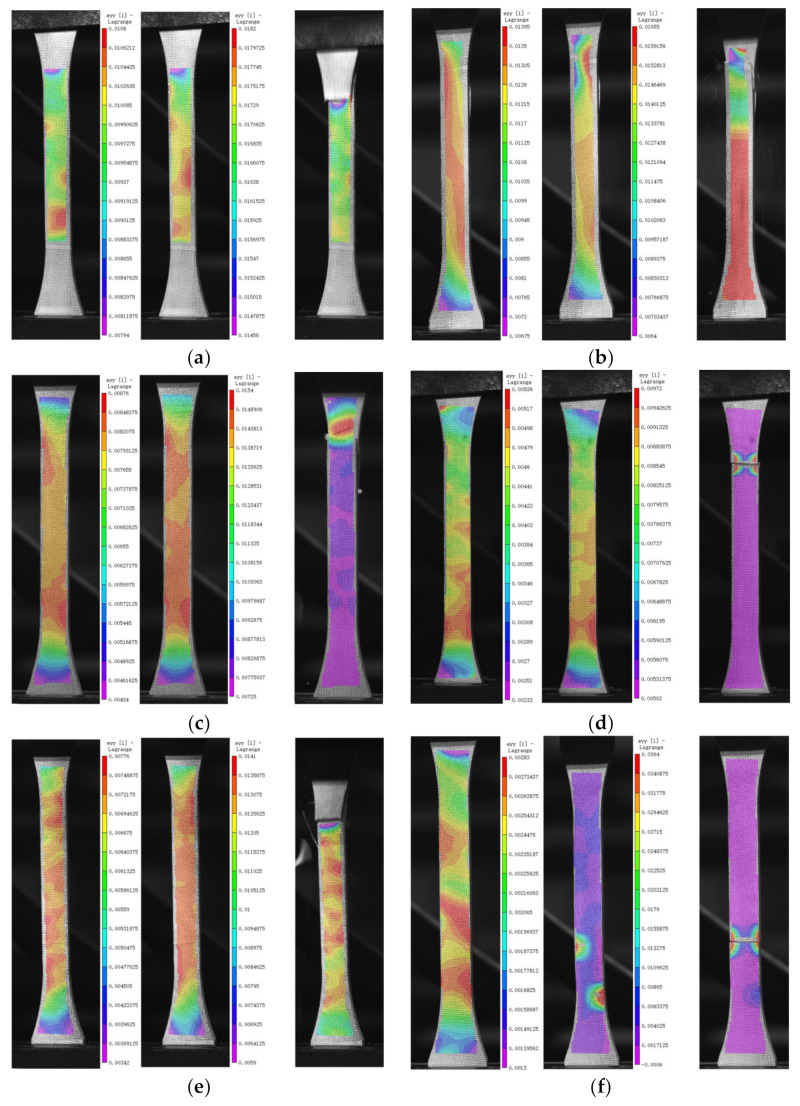

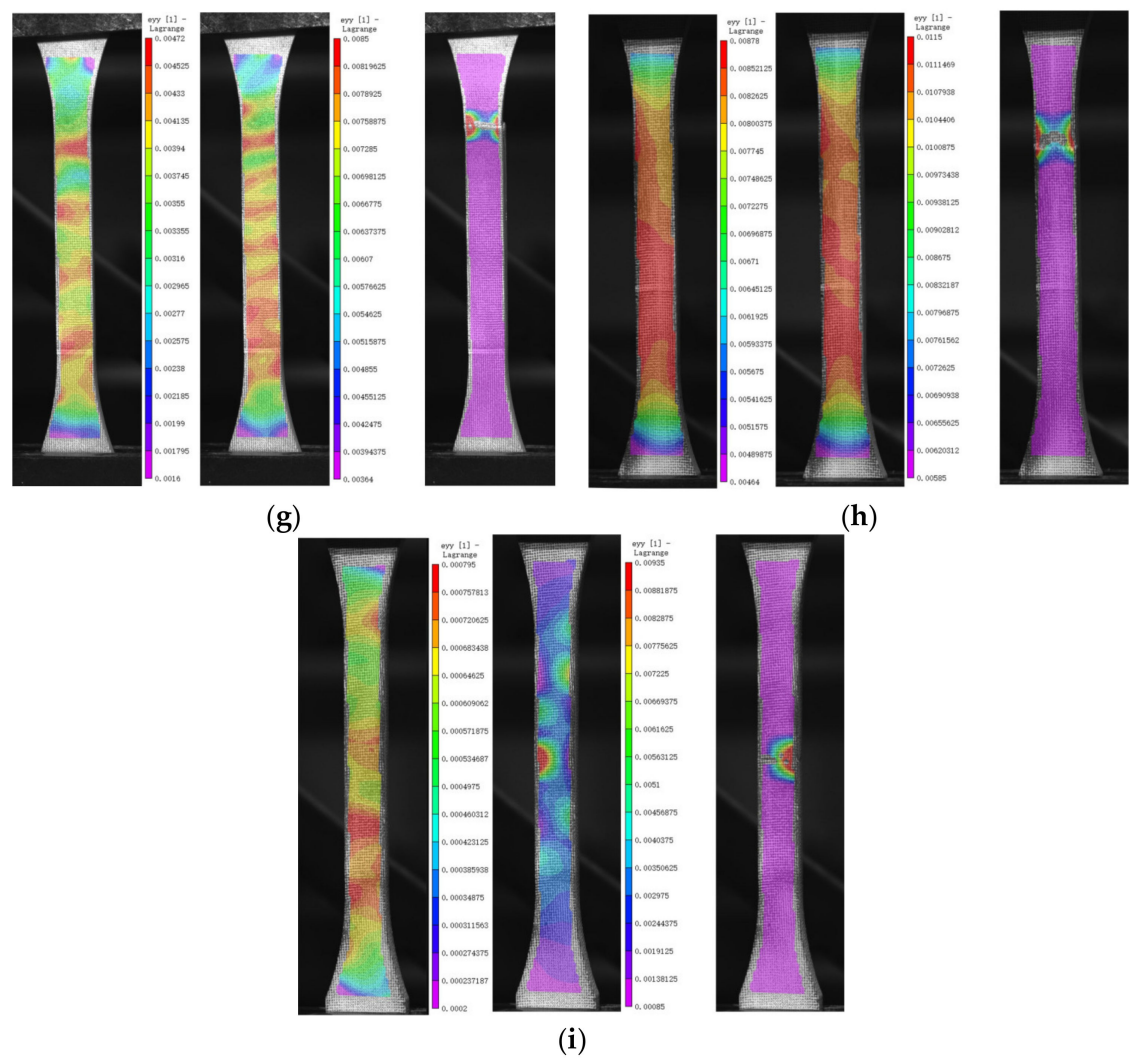
Tensile strain distribution exposed to SW, 5% NaOH, and 5% HCl for 56 d at 20 °C, 50 °C, and 80 °C, respectively. (**a**) SW-20-56, (**b**) 05HA-20-56, (**c**) 05SH-20-56, (**d**) SW-50-56, (**e**) 05HA-50-56, (**f**) 05SH-50-56, (**g**) SW-80-56, (**h**) 05HA-80-56, (**i**) 05SH-80-56.

Figure 11 shows the tensile stress field distribution and fracture location of the specimens after 56 d of exposure to artificial seawater, 5% hydrochloric acid, and a 5% hydroxide nano solution at 20 °C, 50 °C, and 80 °C. The first two figures from the left show the stress field distribution in the middle of the tensile test and when reaching the ultimate load, respectively, while the picture on the right side shows the fracture position of the specimen.

In Figure 11, the failure section of the specimens occurs at the maximum strain, which is in accordance with the maximum tensile strain theory. Furthermore, with the exception of specimens 05SH-50-56 and 05SH-80-56, the ‘maximum strain’ region for all specimens in the exposed condition was close to the clamping end on both sides, with fracture and failure occurring in those locations, which are consistent with Section 3.3.1. In contrast, the 05SH-50-56 and 05SH-80-56 specimens had failure sections in the middle parallel section because the specimens deteriorated to a greater extent in this environment than would be expected from the stress concentration phenomenon. Interestingly, the ‘maximum strain’ region in the specimen is not fixed at a certain place but may shift with the test process and eventually break at the maximum tensile strain, as shown in Figure 11a,f,i. This phenomenon is most evident in 05SH-50-56, where the specimen was the first to reach maximum strain and develop a crack near the lower fixture during tension; however, this was quickly followed by the development of a new crack above the previous, where it fractured completely.

Additionally, the strain fields of the specimens in different environments (with the exception of 05SH-50-56 and 05SH-80-56) were relatively smooth and uniform. This correlates with the degree of material deterioration, and in relation to Section 3.3, the strain field for specimens with higher strength retention is smoother and more homogeneous. In contrast, the strain field distributions for specimens exposed to 05SH-50-56 and 05SH-80-56, which were the most severely weakened in terms of tensile properties, were patchy and discontinuous. This is due to the fact that pultruded GFRP profiles are generally relatively homogeneous. The GFRP which had not been severely eroded also showed relatively homogeneous deformation during tension. However, the distribution of defects within the GFRP, such as micropores, was not completely homogeneous, resulting in a considerable deterioration of the defective localities compared to other locations in an aggressive environment, which would be the first to induce greater strains and subsequently lead to a patchy distribution of the stress field.

### 3.5. SEM Analysis

**Figure 12:**
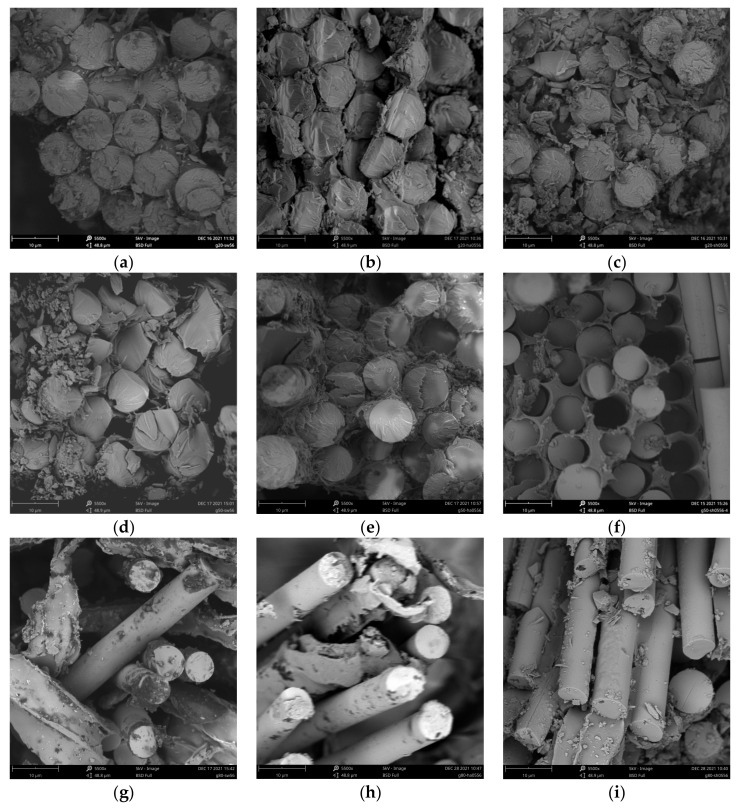
SEM images of fractured sections of specimens. (**a**) SW-20-56, (**b**) 05HA-20-56, (**c**) 05SH-20-56, (**d**) SW-50-56, (**e**) 05HA-50-56, (**f**) 05SH-50-56, (**g**) SW-80-56, (**h**) 05HA-80-56, (**i**) 05SH-80-56.

Scanning electron micrographs of the fracture surfaces of the failed tensile specimens are shown in Figure 12. Comparing the microscopic morphology of the fracture surfaces after 56 d of immersion in SW, 05HA, and 05SH at 20 °C, 50 °C, and 80 °C, it was observed that there was no significant difference in the fracture morphology of the specimens exposed to different chemical environments at 20 °C. The fiber and resin sections were flat and compact, indicating that soaking at 20 °C had a good bonding effect between the fibers and resin, with no significant deterioration. When the ambient temperature was increased to 50 °C, gaps at the resin-glass fiber interface could be observed more clearly, with jagged fracture planes between the fibers. In particular, a large number of holes where the fibers were pulled out existed in the specimen section for the alkali environment. This indicates that, owing to the increased ambient temperature, the bonding between the fibers and resin matrix was substantially reduced, weakening the ability of the fibers to work perfectly with each other. When the immersion temperature was further increased to 80 °C, the bonding of the glass fibers to the epoxy resin in the specimen sections for all three chemical environments was further degraded (especially in the alkaline environment) and the nearly bare fibers, as well as the deboned strips of resin, were clearly visible, indicating that a large amount of resin matrix was withdrawn from the GFRP after 56 d of immersion at 80 °C. These observations support the discussions in Section 3.3.2 (Tensile strength and modulus).

**Figure 13:**
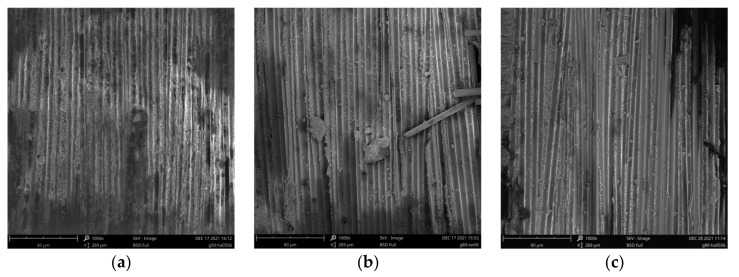
SEM images of the specimens’ strip surfaces. (**a**) 05HA-50-56, (**b**) SW-80-56, (**c**) 05HA-80-56.

Figure 13 illustrates the microscopic morphology of the peeled layer surface of a specimen exhibiting the delamination failure mode. Figure 13a,b clearly show the neatly defined grooves left by the peeled fibers, whereas Figure 13c shows the smooth fiber surface after the resin has been peeled. During the stretching process, the fibers on the surface of the sandwich that comprise the GFRP sheet peel off from the resin between the bonded sandwich, resulting in delamination. The delamination only occurred in specimens exposed to higher temperatures in acidic and seawater environments and at the later stage of the tensile test. A plausible explanation for this is that the resin bond between the interlayers of the specimens in these environments was severely degraded beyond the tensile capacity of the single interlayers, with splitting damage occurring before the specimens were damaged; for specimens in deionized water and alkaline environments, the degradation in tensile properties of the specimens was even more severe, with the tensile strength limit being reached before the specimens became delaminated. In addition, a comparison in Figure 13 shows that the higher the immersion temperature, the smoother the grooves left after the fibers are peeled, indicating more severe resin debonding. This explains the macroscopic phenomenon in Section 3.3.1 that delamination damage is more likely to occur at 80 °C than at 50 °C in the same chemical environment.

### 3.6. Prediction of the Long-Term Behavior and Service Life of GFRP Tensile Strength

Based on the results of the accelerated aging tests, the proposed deterioration model based on the Arrhenius theory [34] was used to predict the long-term tensile properties of the GFRPs. The following assumptions were made using the model: (1) Only one mode of chemical degradation can dominate the deterioration process, and the mode cannot vary with time or temperature. (2) The FRP must deteriorate in an aqueous solution but not in a dry environment [35]. The model considers temperature effects in accelerated aging tests and provides an accurate prediction for materials exposed to temperatures below their glass transition temperatures [36]. In this model, the relationship between performance retention Y and deterioration time t is given by Equation (4).

(4) $$Y=\left(100-Y_{\infty }\right)\exp\left(-\frac{t}{\tau }\right)+Y_{\infty }$$

where Y represents the retention of mechanical properties (%), t is the exposure time, τ is the regression fitting parameter, and $Y_{\infty }$ is the retention of mechanical properties (%) for an infinitely long exposure time. Shenzhen, China, was chosen as the target site for the predictions.

In summary, the prediction procedure for each specimen in different chemical exposure environments consisted of four steps. Step 1 involved fitting the test results using Equation (4) to determine the regression fit parameters τ and $Y_{\infty }$ at different temperatures. The fitting results are presented in Table 3.

**Figure 14:**
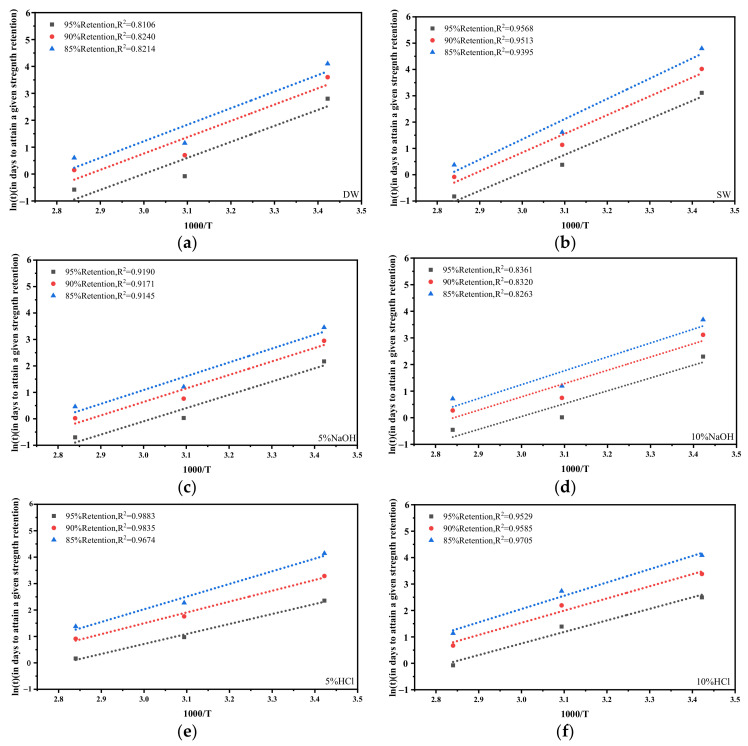
Arrhenius plots: specimens exposed to (**a**) DW, (**b**) SW, (**c**) 5% NaOH, (**d**) 10% NaOH, (**e**) 5% HCl, and (**f**) 10% HCl.

Step 2 was for determining $\frac{E_{a}}{R}$ (i.e., the slope of the Arrhenius curve) of the samples for each exposure environment, where $E_{a}$ is the activation energy, and R is the universal gas constant. To this end, a linear fit of the logarithm (ln(t)) of the time taken for the sample retention (%) to reach a certain value (i.e., 95%, 90%, and 85% in this study) was plotted against 1000/K at different exposure temperatures (where K represents the absolute temperature). ln(t) at different temperatures can be determined from Equation (4) and the fitting parameters obtained in step 1. Figure 14 shows the Arrhenius plots and $\frac{E_{a}}{R}$ values for each sample type. The fitted straight lines for each type are nearly parallel, and the regression lines have R^2^ values of at least 0.8106 (all above 0.80), indicating that the accelerated deterioration test is valid and that the model can be used to predict degradation in the tensile strength of the GFRP. The mean values of these slopes represent $\frac{E_{a}}{R}$ values, with different $\frac{E_{a}}{R}$ values indicating different degradation rates and possibly different degradation mechanisms.

Step 3 was to calculate the time shift factor (TSF) for different exposure conditions using Equation (5).

(5) $$\mathrm{TSF}=\exp\left[\frac{E_{a}}{R}\left(\frac{1}{T_{0}}-\frac{1}{T_{1}}\right)\right]$$

where $T_{0}$ is the lowest temperature (the annual average temperature in Shenzhen, China) and $T_{1}$ is the highest temperature (the exposure temperature in this study). Table 3 lists the TSFs of the six exposure environments.

**Figure 15:**
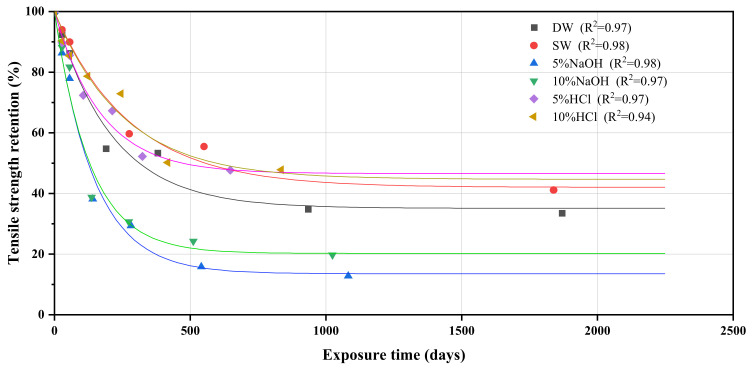
Predicted relationship between residual tensile strength and service life at the temperature of 22.3 °C for the GFRP exposed to various conditions.

In the final step, the TSF was multiplied by the corresponding exposure time at different temperatures to obtain the conversion time, and Equation (4) was used to plot the conversion time against the corresponding tensile strength retention to predict the long-term tensile performance of the GFRP, as shown in Figure 15. R^2^ of the fitted line is at least 0.94, indicating that the Arrhenius model can accurately predict the deterioration pattern of the GFRP. The predicted results are in agreement with the experimental results, and it is evident that most of the performance loss occurs at early stages, regardless of the exposure environment. Tensile properties were predicted to decrease by almost 40% after only 89 d in a 5% sodium hydroxide environment at 22.3 °C, whereas GFRP showed the best resistance to weak-acidic environments, maintaining 49.4% tensile properties after 500 d in a 5% hydrochloric acid environment at 22.3 °C. Because the test environment is a long-time immersion in a solution, the above prediction results may be conservative compared to those of actual working conditions.

## 4. Conclusions

The change in mass of GFRP increases with increasing ambient temperature and immersion time; however, its rate of mass change decreases with time gradually. In deionized water, artificial seawater, and alkaline solutions at 80 °C, the mass change of the GFRP was mainly characterized by an increase in mass; however, in acidic and alkaline solutions at 20 °C and 50 °C, it was mainly characterized by a mass loss.

The tensile strength and stiffness degradation of the GFRP were positively correlated with mass change. The residual tensile strength decreased with increasing ambient temperature and immersion time, but the rate of decrease gradually became slower with time. Compared to deionized water, artificial seawater, and acid, the alkaline environment had the greatest effect on the degradation of GFRP properties, with only 12.60% and 19.61% of the residual tensile strength after 56 d of exposure to 05SH-80 and 10SH-80, respectively, with degradation of tensile strength being more severe than that of tensile stiffness for GFRP.

The SEM results show that fiber-matrix debonding is most significant when the GFRP is exposed to alkaline environments, whereas the interlayer fibers of the GFRP are completely exposed in acidic and seawater environments at higher temperatures.

It can be deduced from the DIC image analysis that, at 50 °C and 80 °C, the GFRP corrodes uniformly when exposed to deionized water and acids but unevenly when exposed to alkaline solutions. The location of maximum strain may shift during tension, but failure nearly always occurs at the maximum strain.

The Arrhenius model was validated using experimental data, demonstrating that the model has good applicability. Compared to artificial seawater and alkaline environments, the GFRP is better at resisting long-term erosion in acidic environments. The tensile residual strength of the GFRP was predicted, using the Arrhenius model, to be 46.48% and 44.62% after exposure to 5% and 10% hydrochloric acid, respectively, for 2000 d in Shenzhen. The above test results and long-term performance prediction are of reference significance for the application of GFRP in Shenzhen.

## Figures and Tables

**Table 1:** Experimental scheme.

Groups	Conditioning Environment	Immersion Medium	TEMP (°C)	Age (d)
1	DW-20	Deionized water	20	28, 56
2	DW-50		50	
3	DW-80		80	
4	SW-20	Sea water	20	28, 56
5	SW-50		50	
6	SW-80		80	
7	05HA-20	5% HCl solution	20	28, 56
8	05HA-50		50	
9	05HA-80		80	
10	10HA-20	10% HCl solution	20	28, 56
11	10HA-50		50	
12	10HA-80		80	
13	05SH-20	5% NaOH solution	20	28, 56
14	05SH-50		50	
15	05SH-80		80	
16	10SH-20	10% NaOH solution	20	28, 56
17	10SH-50		50	
18	10SH-80		80	

**Table 2:** Summary of mechanical test results.

Conditioning			Tensile Strength			Modulus of Elasticity		
Exp.	T	Age	Avg	COV	Retention	Avg	COV	Retention
	(°C)	(Week)	(MPa)	(%)	(%)	(GPa)	(%)	(%)
REF	20	-	407.9	5.3	100	30.6	0.0	100
DW	20	4	384.8	0.8	94	29.9	2.8	98
		8	351.6	1.6	86	29.1	5.2	95
	50	4	223.4	6.6	55	28.8	1.2	94
		8	217.5	3.2	53	27.6	2.8	90
	80	4	141.8	1.6	35	25.5	0.2	83
		8	136.4	2.0	33	25.0	1.2	82
SW	20	4	383.4	3.8	94	29.3	1.7	96
		8	367.0	2.2	90	27.8	0.4	91
	50	4	243.2	3.9	60	28.5	0.4	93
		8	226.0	3.3	55	26.8	2.5	88
	80	4	167.4	6.8	41	26.1	1.7	85
		8	166.5	4.6	41	25.2	1.8	82
5% SH	20	4	352.2	2.8	86	29.8	2.4	98
		8	317.8	2.7	13	29.4	1.1	96
	50	4	155.3	4.6	38	29.8	5.1	98
		8	119.4	3.3	29	28.4	0.9	93
	80	4	63.8	8.6	16	20.5	3.1	67
		8	51.4	5.0	13	17.2	3.9	56
10% SH	20	4	359.9	0.8	88	29.2	0.5	96
		8	333.3	4.0	82	29.2	0.6	96
	50	4	157.7	7.4	39	29.0	2.6	95
		8	124.7	1.1	31	28.3	0.5	92
	80	4	98.4	4.0	24	20.1	4.9	66
		8	80.0	9.4	20	17.6	3.6	57
5% HA	20	4	365.9	4.2	90	28.9	0.7	94
		8	349.1	1.3	86	28.4	2.4	93
	50	4	295.2	2.2	72	29.1	4.8	95
		8	274.3	3.1	67	27.1	1.1	89
	80	4	212.7	3.8	52	22.5	0.4	74
		8	194.2	3.8	48	22.4	0.6	73
10% HA	20	4	368.5	1.5	90	29.4	1.9	96
		8	348.4	1.8	85	28.9	1.8	94
	50	4	320.9	4.0	79	28.6	2.3	93
		8	297.4	1.8	73	26.8	0.7	88
	80	4	204.5	3.0	50	22.1	1.8	72
		8	194.7	3.1	48	20.8	0.6	68

**Table 3:** Regression coefficients in Equation (4) and time shift factors (TSF) for the GFRP specimens.

Conditioning	TEMP (°C)	τ	Y_∞_	Ea/R	TSF (Shenzhen-22.3 °C)
DW	20	125.93	61.53	6054	0.85
	50	8.13	53.26	6054	5.79
	80	7.18	33.42	6054	28.44
SW	20	70.11	81.75	7222	0.83
	50	12.41	54.89	7222	8.13
	80	4.96	40.81	7222	54.25
5% NaOH	20	58.05	64.30	5111	0.87
	50	14.35	27.81	5111	4.41
	80	8.43	12.49	5111	16.89
10% NaOH	20	47.44	73.58	5015	0.88
	50	13.82	29.33	5015	4.28
	80	9.92	19.33	5015	16.02
5% HCl	20	30.71	82.79	4226	0.89
	50	16.61	66.06	4226	3.41
	80	11.88	47.14	4226	10.50
10% HCl	20	41.37	80.33	4665	0.88
	50	21.39	70.76	4665	3.87
	80	9.23	47.61	4665	13.19

## Data Availability

The data supporting this study are available from the corresponding author upon reasonable request.
